# Author Correction: Farnesoid X receptor activation inhibits TGFBR1/TAK1-mediated vascular inflammation and calcification via miR-135a-5p

**DOI:** 10.1038/s42003-020-01414-1

**Published:** 2020-11-06

**Authors:** Chao Li, Shijun Zhang, Xiaoqing Chen, Jingkang Ji, Wenqing Yang, Ting Gui, Zhibo Gai, Yunlun Li

**Affiliations:** 1 Experimental Center, Shandong University of Traditional Chinese Medicine, 250355 Jinan, China; 2 Department of Clinical Pharmacology and Toxicology, University Hospital Zurich, 8032 Zurich, Switzerland; 3 Affiliated Hospital of Shandong University of Traditional Chinese Medicine, 250000 Jinan, China

**Keywords:** Cell biology, Drug discovery

Correction to: *Communications Biology* 10.1038/s42003-020-1058-2, published online 24 June 2020.

In the original version of the published article, the image shown in Fig. 1h labelled as Pi + Ca (TGFBR1 siRNA) was incorrect. The image has been replaced with the correct photograph in the HTML and PDF versions of the article. The incorrect figure panel is reproduced here.

**Figure 1:**
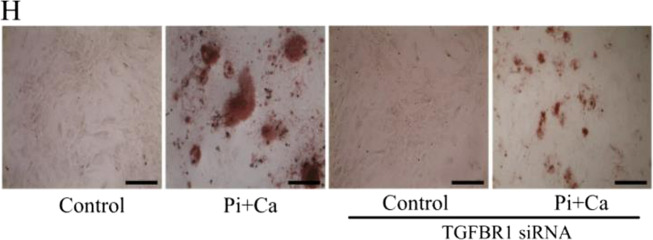
Fig. 1

