# Supplementary material for: Farnesoid X receptor activation inhibits TGFBR1/TAK1-mediated vascular inflammation and calcification via miR-135a-5p
Source: Commun Biol. 2020 Jun 24;3:327. doi: 10.1038/s42003-020-1058-2 (PMC7314757; doi:10.1038/s42003-020-1058-2)
Supplement: Supplementary file 1 — Supplementary Information [file 42003_2020_1058_MOESM1_ESM.pdf]

Supplementary Figures

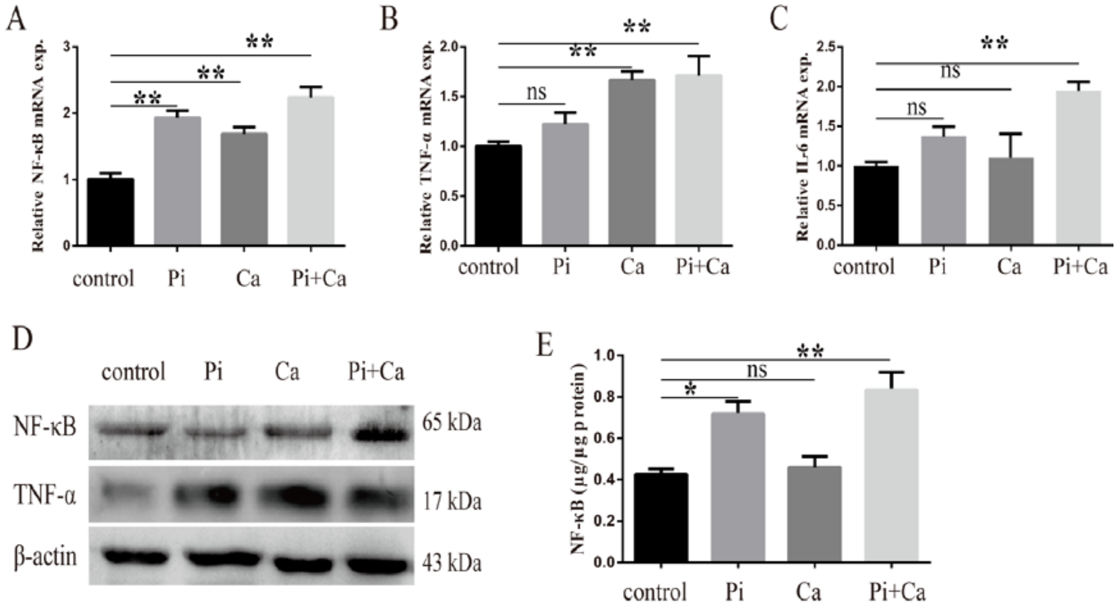

Supplemental Figure 1. Osteogenic medium increased the expression of proinflammatory cytokines in HASMCs. HASMCs were cultured with osteogenic medium containing 2.0 mM Pi and 2.7 mM Ca for 7 days. **A-C**, The mRNA expression of proinflammatory cytokines (NF-κB, TNF-α, and IL-6) was increased in osteogenic medium-cultured HASMCs. **D**, Western blot showing NF-κB and TNF-α protein expression were stimulated by osteogenic medium. **E**, Increased intracellular NF-κB content in osteogenic medium-cultured cells was assayed by NF-κB p65 ELISA kit. *$P<0.05$ versus control group, **$P<0.01$ versus control group, ns indicates no significant versus control group. Data were pooled as mean±S.D. (error bars) from three or more independent experiments.

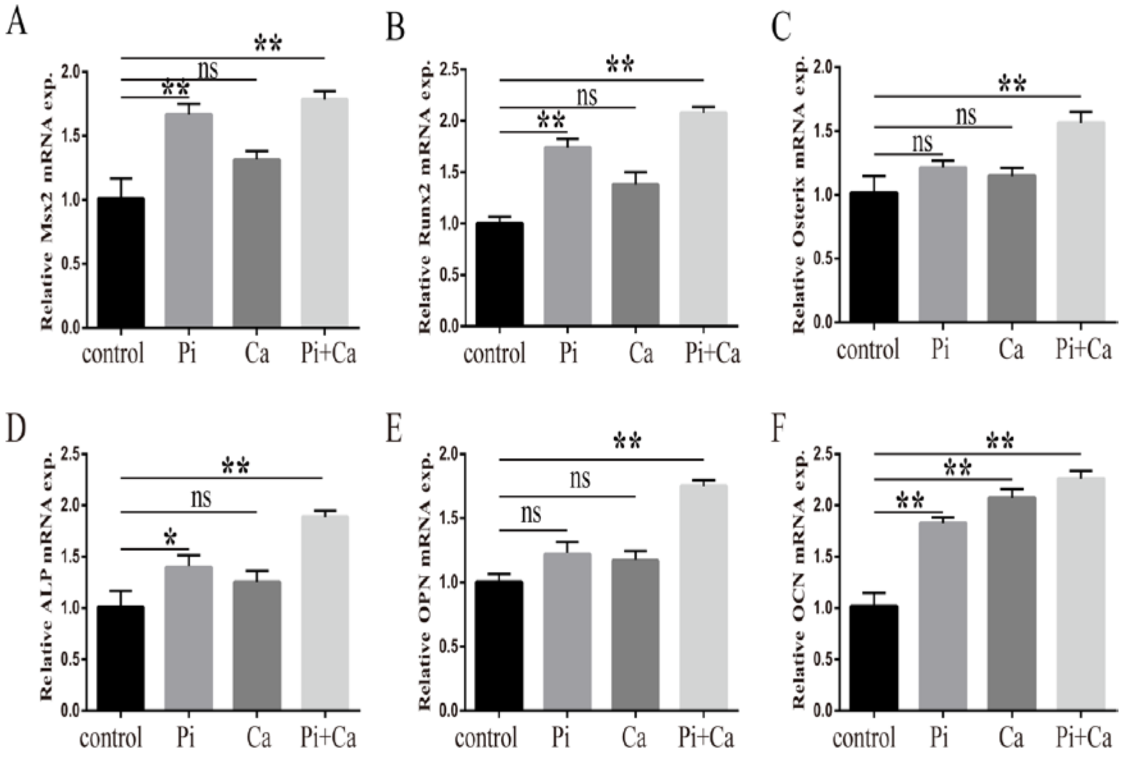

Supplemental Figure 2. Osteogenic medium increased the mRNA expression of osteogenic transcription factors and osteogenic markers in HASMCs. HASMCs were cultured with osteogenic medium containing 2.0 mM Pi and 2.7 mM Ca for 7 days. The mRNA expression of osteogenic transcription factors (Msx2, Runx2 and osterix) **(A-C)** and osteogenic markers (ALP, OPN and OCN) **(D-E)** were found to be increased in osteogenic medium-cultured HASMCs. *P<0.05 versus control group, **P<0.01 versus control group, ns indicates no significant versus control group. Data were pooled as mean±S.D. (error bars) from three or more independent experiments.

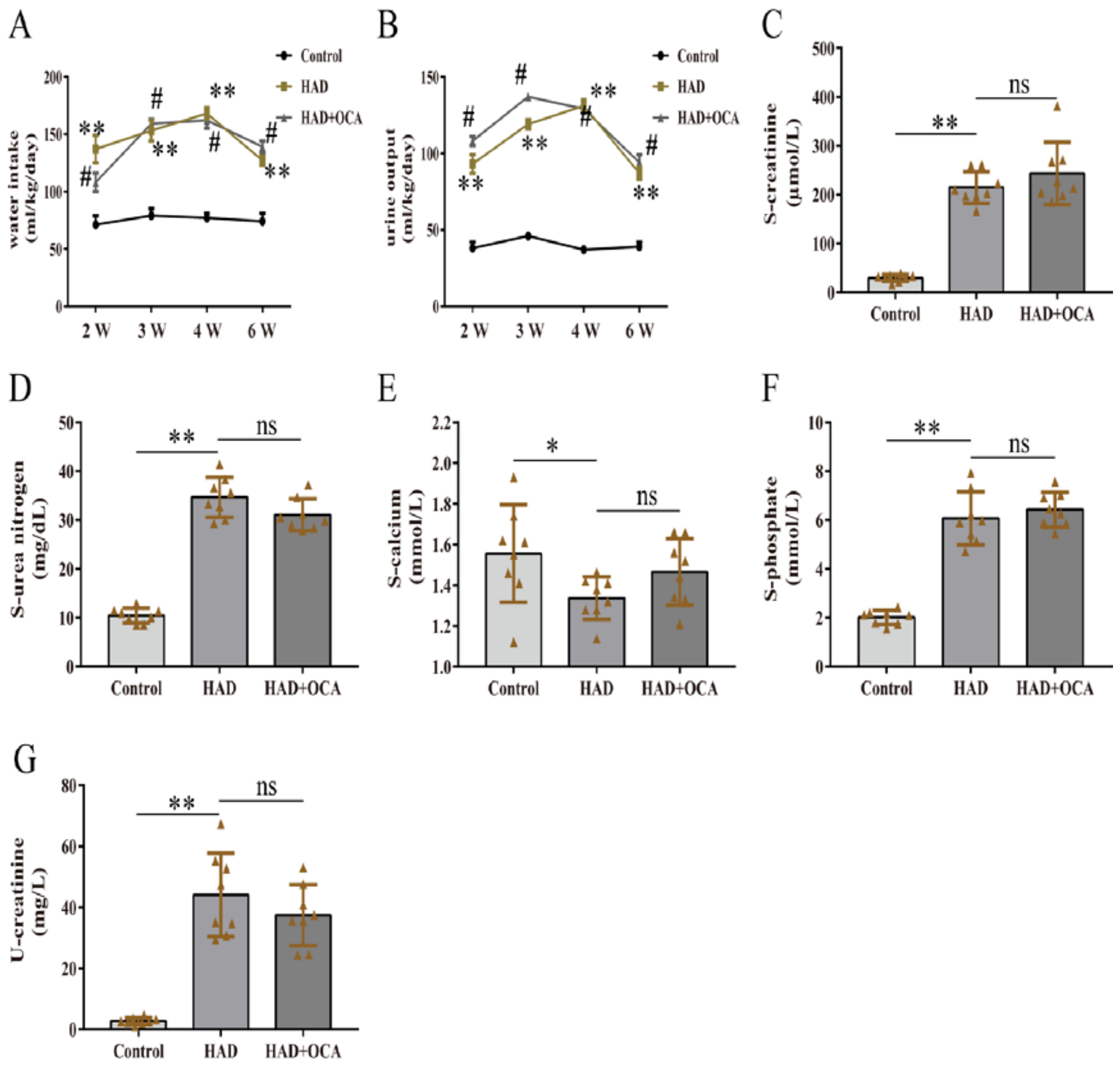

Supplemental Figure 3. Kidney function of rats which were fed with high adenine diet in the absence or presence of OCA. After feeding with high adenine diet in the absence or presence of OCA for 4 weeks, kidney function of rats were measured (n=8 per group). Water intake **(A)** and urine output **(B)** of rats in HAD group and HAD+OCA group were increased compared with control group rats, but there is no significant difference between HAD group and HAD+OCA group. **P<0.01 versus control group, #P>0.05 versus HAD group. The serum concentration of creatinine **(C)**, urea nitrogen **(D)**, and phosphate **(F)** were also increased in HAD group and HAD+OCA group, and there is no significant difference between HAD group and HAD+OCA group. **E,** Calcium concentration in serum was decreased in rats after feeding with high adenine diet, and OCA have no effect on regulating calcium concentration. **G**, Creatinine in urine was also increased in HAD group and HAD+OCA group, and difference was not statistically significant between HAD group and HAD+OCA group. *P<0.05 versus control group, **P<0.01 versus control group, ns indicates no significant versus HAD group.

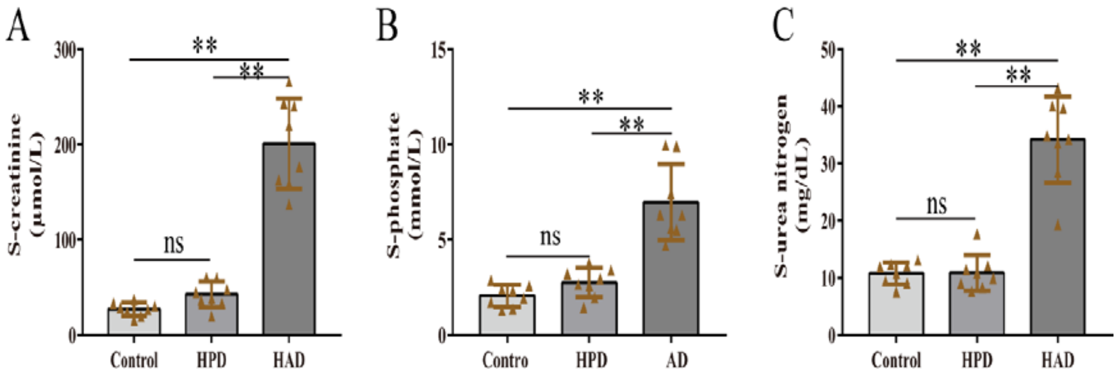

Supplemental Figure 4. Biochemical detection of rats which were fed with high phosphate diet in the absence or presence of adenine. After feeding with high phosphate diet in the absence or presence of adenine for 4 weeks, Biochemical detection of rats were measured (n=8 per group). High-adenine diet (HAD) is based on HPD plus adenine to induce chronic kidney disease and vascular calcification. Therefore, HPD was established as a dietary control group of HAD. The serum concentration of creatinine **(A)**, urea nitrogen **(B)**, and phosphate **(C)** were increased in high-adenine diet (HAD group), while this was not the case in rats fed with a high-phosphate diet (HPD). **P<0.01 versus control group, ns indicates no significant versus control group.

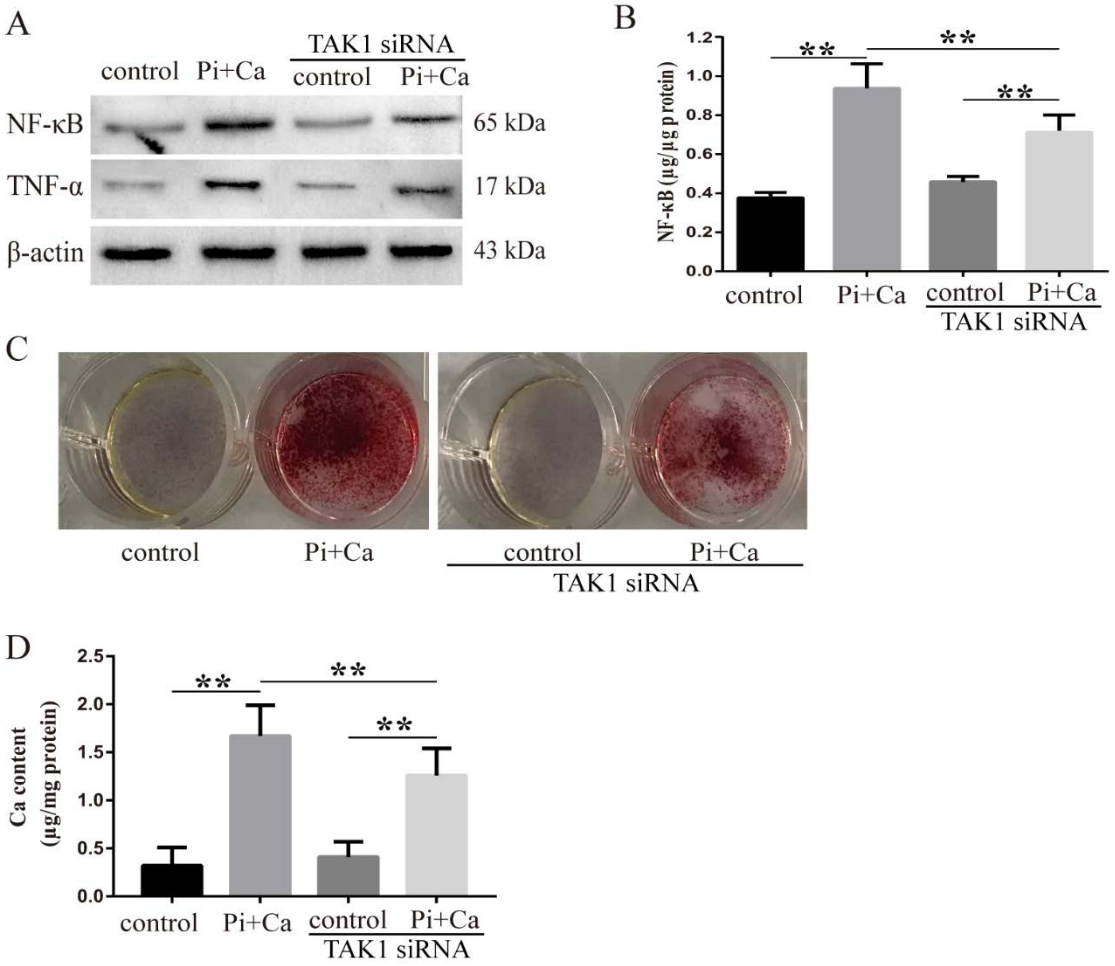

Supplemental Figure 5. TAK1 knockdown attenuated osteogenic medium-induced HASMCs inflammation and calcification. A, Western blot analysis of the expression of NF-κB and TNF-α in osteogenic medium-treated HASMCs in the absence or presence of TAK1 siRNA. **B**, NF-κB content in indicated groups was assayed by NF-κB p65 ELISA kit. **C**, Representative images of alizarin red staining in indicated groups. **D**, Calcium content of HASMCs in indicated groups. $^{**}P<0.01$. Data were pooled as mean±S.D. (error bars) from three or more independent experiments.

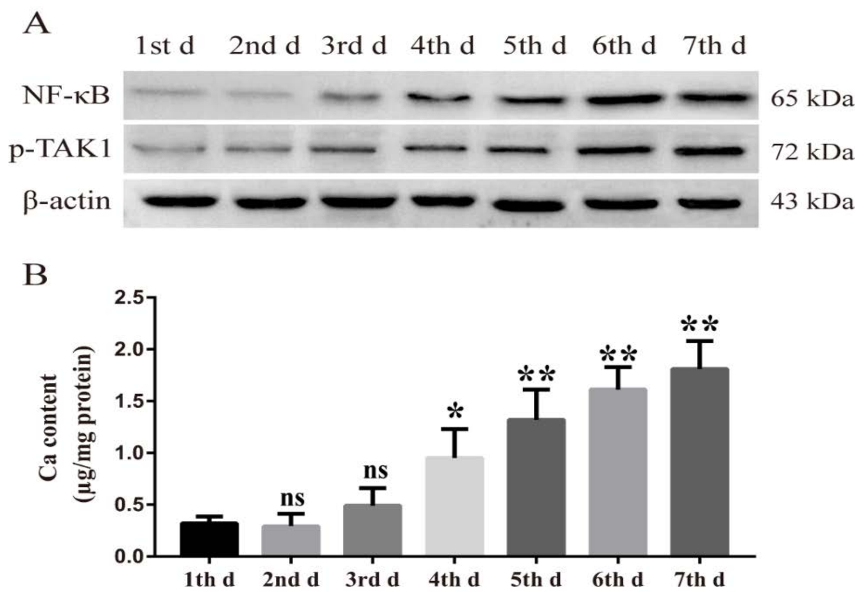

Supplemental Figure 6. Time course during 7 days of the expression of p-TAK1 and NF-κB in osteogenic medium-cultured HASMCs. A, Western blot analysis of the expression of p-TAK1 and NF-κB in the cells of each day after treating with osteogenic medium. **B**, Calcium content of HASMCs in the cells of each day after treating with osteogenic medium was measured. *P<0.05 versus 1th d, **P<0.01 versus 1th d, ns indicates no significant versus 1th d. Data were pooled as mean±S.D. (error bars) from three or more independent experiments.

Uncropped blot images

Figure 1-D

**TAK1**	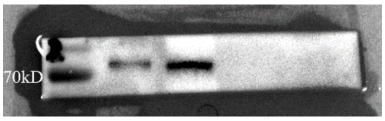	**p-TAK1**	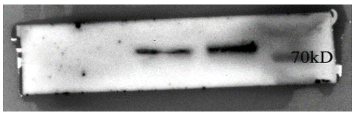
**TAB1**	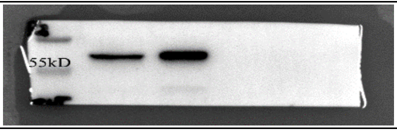	**p-IκBα**	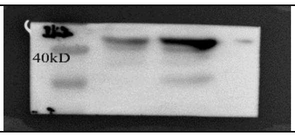
**β-actin**	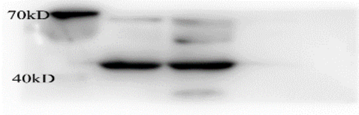		

Figure 1-E

**NF-κB**	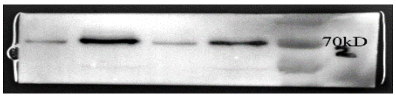	**TNF-α**	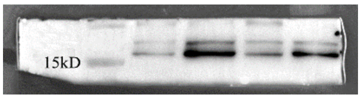
**TAK1**	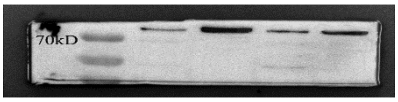	**p-TAK1**	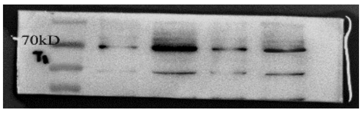
**TAB1**	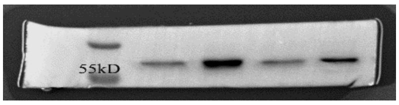	**p-IκBα**	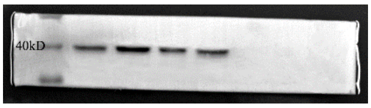
**β-actin**	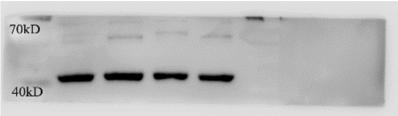		

Figure 2-F

**NF-κB**	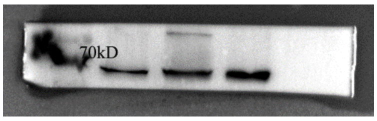	**TNF-α**	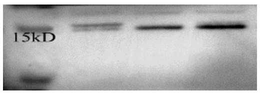
**β-actin**	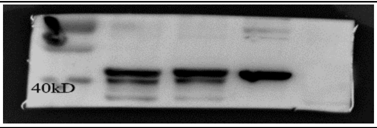		

Figure 2-G

**TAK1**	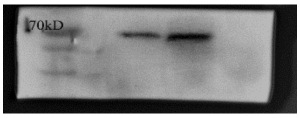	**p-TAK1**	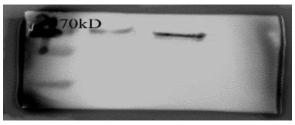

**TAB1**	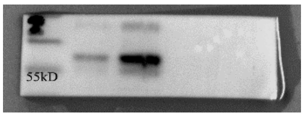	**p-IκBα**	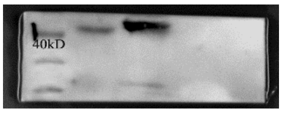
**β-actin**	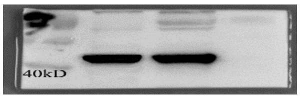		

Figure 3-A

**FXR**	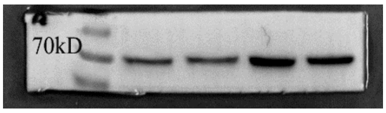	**β-actin**	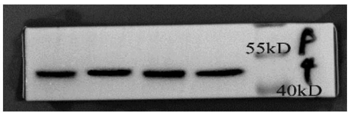

Figure 3-B

**NF-κB**	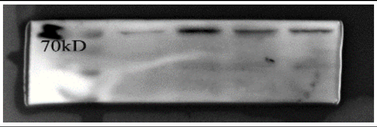	**TNF-α**	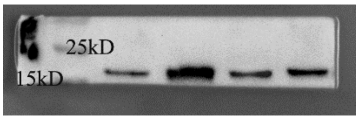
**TAK1**	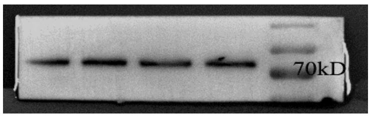	**p-TAK1**	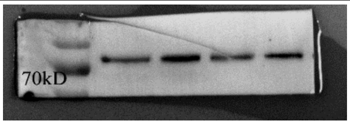
**TAB1**	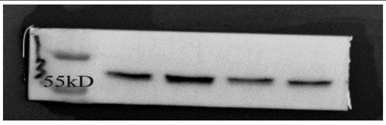	**p-IκBα**	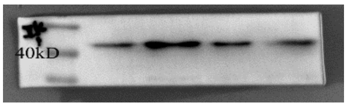
**β-actin**	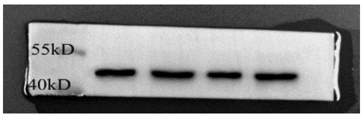		

Figure 4-E

**NF-κB**	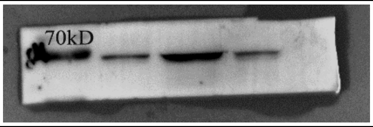	**TNF-α**	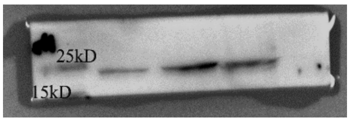
**β-actin**	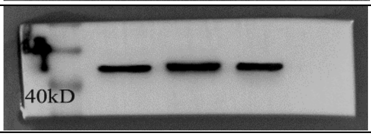		

Figure 5-F

**NF-κB**	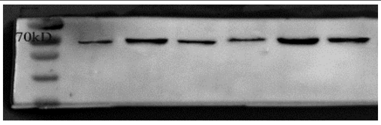	**TNF-α**	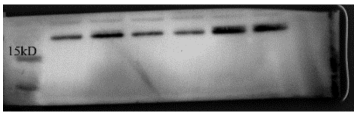

**β-actin**	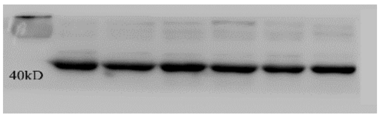		

Figure 6-C

**TAK1**	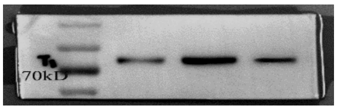	**p-TAK1**	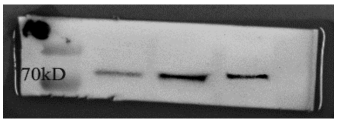
**TAB1**	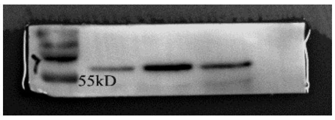	**p-IκBα**	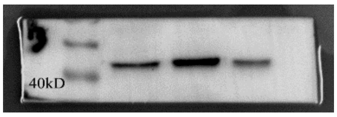
**β-actin**	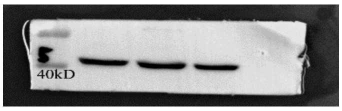		

Figure 6-E

**TAK1**	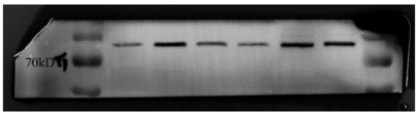	**p-TAK1**	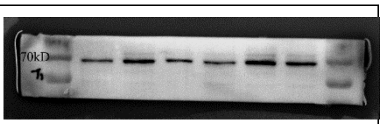
**TAB1**	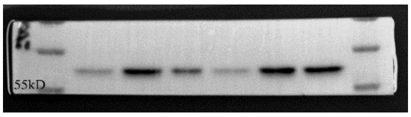	**p-IκBα**	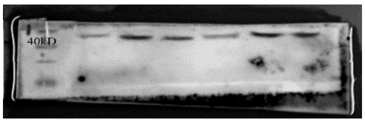
**β-actin**	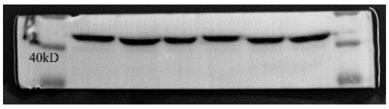		

Figure 7-F

**NF-κB**	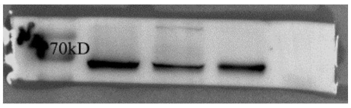	**TNF-α**	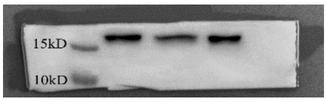
**TAK1**	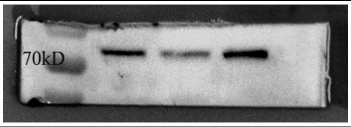	**p-TAK1**	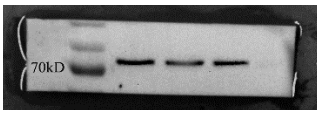
**TAB1**	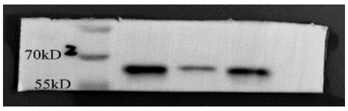	**p-IκBα**	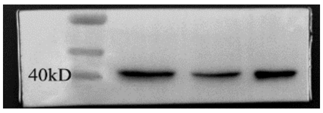
**β-actin**	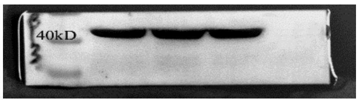		

Supplementary Figure 1-D

**NF-κB**	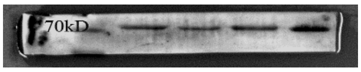	**TNF-α**	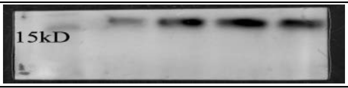
**β-actin**	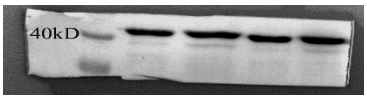		

Supplementary Figure 5-A

**NF-κB**	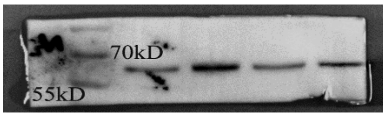	**TNF-α**	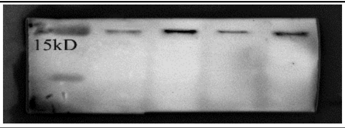
**β-actin**	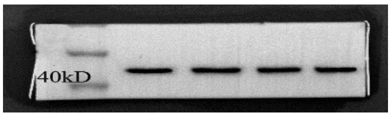		

Supplementary Figure 6-A

**NF-κB**	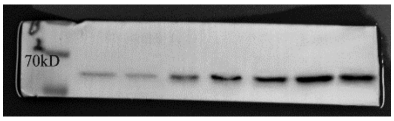	**p-TAK1**	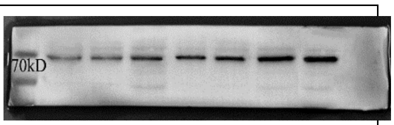
**β-actin**	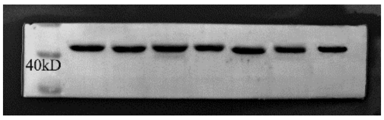		
