# Supplementary material for: Farnesoid X receptor activation inhibits TGFBR1/TAK1-mediated vascular inflammation and calcification via miR-135a-5p
Source: Commun Biol. 2020 Jun 24;3:327. doi: 10.1038/s42003-020-1058-2 (PMC7314757; doi:10.1038/s42003-020-1058-2)
Supplement: Supplementary file 3 — Reporting Summary [file 42003_2020_1058_MOESM3_ESM.pdf]

nature research

Corresponding author(s): Yunlun Li, Zhibo Gai

Last updated by author(s): May 27, 2020

Reporting Summary

Nature Research wishes to improve the reproducibility of the work that we publish. This form provides structure for consistency and transparency in reporting. For further information on Nature Research policies, see Authors & Referees and the Editorial Policy Checklist .

Statistics

For all statistical analyses, confirm that the following items are present in the figure legend, table legend, main text, or Methods section.

n/a	Confirmed	
☐	☒	The exact sample size (*n*) for each experimental group/condition, given as a discrete number and unit of measurement
☐	☒	A statement on whether measurements were taken from distinct samples or whether the same sample was measured repeatedly
☐	☒	The statistical test(s) used AND whether they are one- or two-sided<br>*Only common tests should be described solely by name; describe more complex techniques in the Methods section.*
☐	☒	A description of all covariates tested
☐	☒	A description of any assumptions or corrections, such as tests of normality and adjustment for multiple comparisons
☐	☒	A full description of the statistical parameters including central tendency (e.g. means) or other basic estimates (e.g. regression coefficient) AND variation (e.g. standard deviation) or associated estimates of uncertainty (e.g. confidence intervals)
☐	☒	For null hypothesis testing, the test statistic (e.g. *F*, *t*, *r*) with confidence intervals, effect sizes, degrees of freedom and *P* value noted<br>*Give P values as exact values whenever suitable.*
☐	☒	For Bayesian analysis, information on the choice of priors and Markov chain Monte Carlo settings
☐	☒	For hierarchical and complex designs, identification of the appropriate level for tests and full reporting of outcomes
☐	☒	Estimates of effect sizes (e.g. Cohen's *d*, Pearson's *r*), indicating how they were calculated

Our web collection on statistics for biologists contains articles on many of the points above.

Software and code

Policy information about availability of computer code

Data collection	no software was used.
Data analysis	GraphPad Prism 7, Adobe Illstrator CS6

For manuscripts utilizing custom algorithms or software that are central to the research but not yet described in published literature, software must be made available to editors/reviewers. We strongly encourage code deposition in a community repository (e.g. GitHub). See the Nature Research guidelines for submitting code & software for further information.

Data

Policy information about availability of data

All manuscripts must include a data availability statement. This statement should provide the following information, where applicable:

- Accession codes, unique identifiers, or web links for publicly available datasets
- A list of figures that have associated raw data
- A description of any restrictions on data availability

The data that support the findings of this study are available from the corresponding author upon reasonable request.

Field-specific reporting

Please select the one below that is the best fit for your research. If you are not sure, read the appropriate sections before making your selection.

☒ Life sciences ☐ Behavioural & social sciences ☐ Ecological, evolutionary & environmental sciences

For a reference copy of the document with all sections, see nature.com/documents/nr-reporting-summary-flat.pdf

Life sciences study design

All studies must disclose on these points even when the disclosure is negative.

Sample size	For animal study, n=8 per group. For cells study in vitro, Data were pooled as mean±S.D. (error bars) from three or more independent experiments.
Data exclusions	No
Replication	Confirm
Randomization	Random
Blinding	This study does not involve clinical trials.

Reporting for specific materials, systems and methods

We require information from authors about some types of materials, experimental systems and methods used in many studies. Here, indicate whether each material, system or method listed is relevant to your study. If you are not sure if a list item applies to your research, read the appropriate section before selecting a response.

Materials & experimental systems

n/a	Involved in the study	
☐	☒	Antibodies
☐	☒	Eukaryotic cell lines
☒	☐	Palaeontology
☐	☒	Animals and other organisms
☒	☐	Human research participants
☒	☐	Clinical data

Methods

n/a	Involved in the study	
☒	☐	ChIP-seq
☒	☐	Flow cytometry
☒	☐	MRI-based neuroimaging

Antibodies

Antibodies used

NF-κB antibodies (1:1000, ab32536, Rabbit monoclonal [E379], Abcam),
TNF-α antibodies (1:1000, ab6671, Rabbit polyclonal, Abcam),
FXR antibodies (1:500, sc-13063, Rabbit IgG, Santa Cruz Biotechnology),
TAK1 antibodies (1:1000, ab109526, Rabbit monoclonal [EPR5984], Abcam),
Phospho-TAK1 antibodies(1:1000, ab109404, Rabbit monoclonal [EPR2863], Abcam),
TAB1 antibodies (1:1000, ab227210, Rabbit polyclonal, Abcam),
Phospho-IκBα antibodies (Ser32/36) (1:1000, #9246, Mouse IgG1, Cell Signaling Technology).

Validation

NF-κB antibodies (Host species: Rabbit. Tested applications: WB, IHC-P, ICC/IF, IP)
TNF-α antibodies (Host species: Rabbit. Tested applications: ELISA, IHC-P, WB, ICC/IF)
FXR antibodies (Host species: Rabbit. Tested applications: WB, IP, IF, IHC(P), ELISA)
TAK1 antibodies (Host species: Rabbit. Tested applications: WB, IHC-P, ICC/IF, Flow Cyt)
Phospho-TAK1 antibodies (Host species: Rabbit. Tested applications: Dot blot, WB, IP)
TAB1 antibodies (Host species: Rabbit. Tested applications: ICC/IF, WB)
Phospho-IκBα antibodies (Host species: Mouse. Tested applications: WB)

Eukaryotic cell lines

Policy information about cell lines

Cell line source(s)	Human aortic smooth muscle cells (HASMCs) were purchased from Sciencell (#6110, San Diego, California, USA)
Authentication	Product Name: Human Aortic Smooth Muscle Cells (HASMC). α-smooth muscle actin (SMA): Positive. HIV-1 DNA by PCR: Not detected. HBV DNA by PCR: Not detected. HCV DNA by PCR: Not detected. Mycoplasma DNA by PCR: Not detected. Fungi & Yeast by culture: Negative. Bacteria by culture: Negative. Approved by Hannah Steele (Quality Control).
Mycoplasma contamination	Confirm that cell line tested negative for mycoplasma contamination.
Commonly misidentified lines (See ICLAC register)	NO

Animals and other organisms

Policy information about studies involving animals; ARRIVE guidelines recommended for reporting animal research

Laboratory animals	Wistar rats, 10-week-old, male and female.
Wild animals	This study did not involve wild animals.
Field-collected samples	This study did not involve samples from the field.
Ethics oversight	The animal experiments were performed in accordance with the Guide for the Care and Use of Laboratory Animals (published by the US National Institutes of Health) and were approved by the Institutional Animal Care and Research Advisory Committee of the Shandong University of Traditional Chinese Medicine.

Note that full information on the approval of the study protocol must also be provided in the manuscript.
